# Fission yeast Dap1 heme iron-coordinating residue Y83 is required for cytochromes P450 function

**DOI:** 10.17912/micropub.biology.000631

**Published:** 2022-08-23

**Authors:** Shan Zhao, Adam L Hughes, Peter J Espenshade

**Affiliations:** 1 Johns Hopkins University School of Medicine, USA

## Abstract

Fission yeast Dap1 is a heme binding protein required for cytochromes P450 activity. Here, we tested whether Dap1 axial coordination of heme iron is required for its role in the function of the cytochrome P450 enzymes, Erg5 and Erg11. Two different *dap1* mutants predicted to alter iron coordination failed to rescue growth on cobalt chloride containing medium which requires Erg5 and Erg11. In addition, deletion of *dap1 ^+^* did not affect expression of Erg5 or Erg11. PGRMC1, a mammalian Dap1 homolog, does not require heme binding to bind and stabilize cytochromes P450. These experiments highlight important functional differences between these conserved proteins.

**Figure 1. Fission yeast Dap1 heme iron-coordinating residue Y83 is required for cytochromes P450 function.**
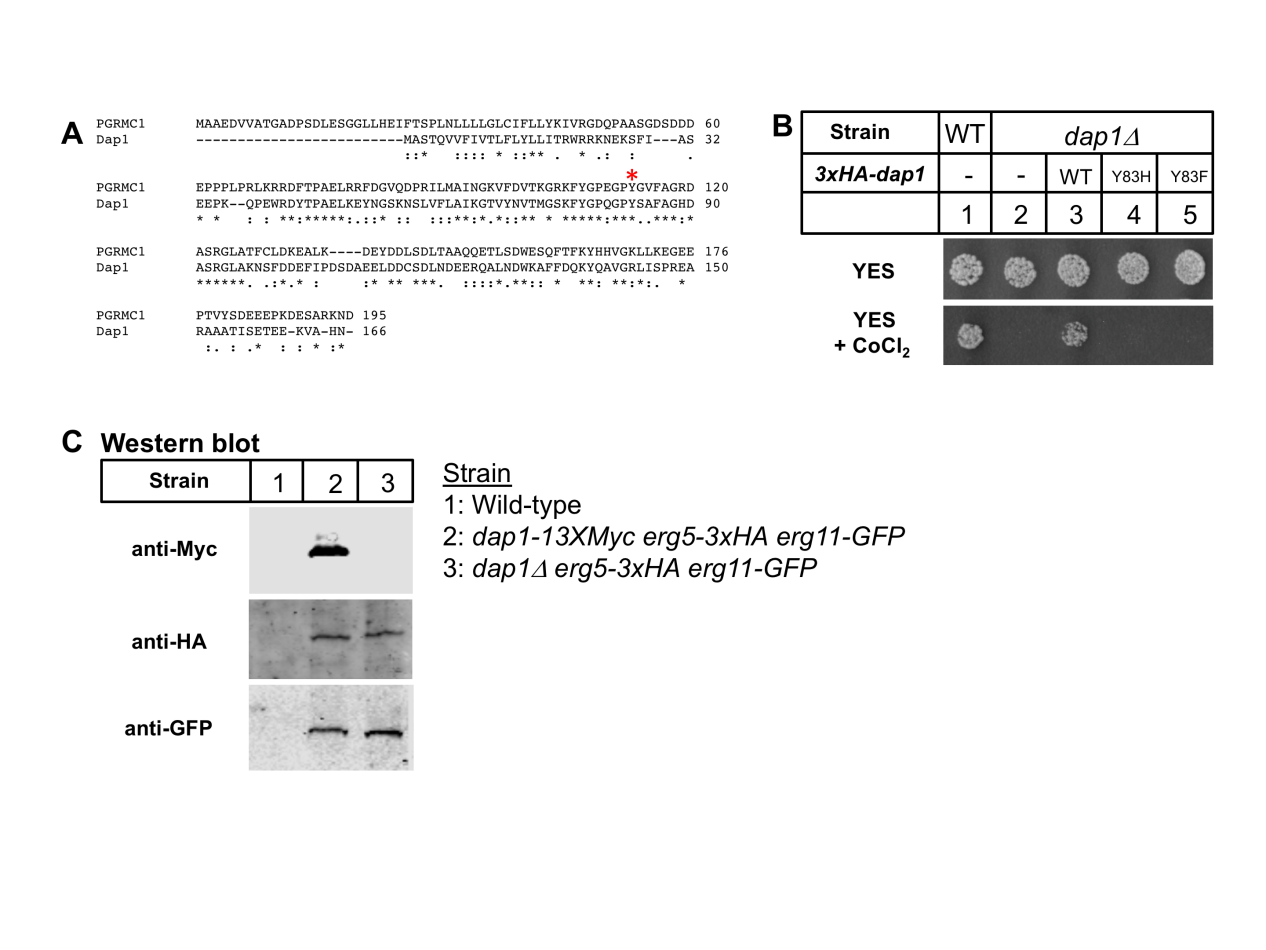
(A) CLUSTAL alignment of human PGRMC1 (195 aa) and *S. pombe* Dap1 (166 aa) showing protein homology. Iron coordinating tyrosine is indicated (PGRMC1 Y113 and Dap1 Y83; red asterisk). (B) Wild-type (KGY425) or *dap1Δ* (PEY901) strains containing either empty vector or *dap1* plasmid were plated on either rich medium (YES) or rich medium containing Cobalt chloride (1.6 mM) and grown at 30 ^o^ C for 3 or 5 days, respectively. (C) Whole cell lysates from the indicated strains (KGY425, PEY1070, PEY1072) were immunoblotted using the antibodies shown.

## Description

Fission yeast Dap1 is a membrane-bound, heme binding protein that binds directly to both fission yeast cytochromes P450, Erg5 and Erg11, and is required for Erg5 and Erg11 function in ergosterol synthesis (Hughes et al., 2007). Progesterone receptor membrane component 1 (PGRMC1) is a mammalian homolog of Dap1 that is also required for cytochromes P450 activity (Hughes et al., 2007; Mallory et al., 2005; McGuire et al., 2021). In mouse liver, PGRMC1 binds more than 13 cytochromes P450 and supports their stability and consequently P450 activity (McGuire et al., 2021). Interestingly, PGRMC2, a paralog of PGRMC1, also binds heme and functions as a heme chaperone required to deliver heme from the mitochondrion to the nucleus in mice (Galmozzi et al., 2019). Whether PGRMC1 also functions as a heme chaperone and what specific role PGRMC1 heme binding serves are unknown.

PGRMC1 contains a cytochrome *b5-* like domain (Cahill, 2007). Cytochrome *b _5_* binds to heme, and its heme iron is six-coordinated (Schenkman and Jansson, 2003). Structural studies of the PGRMC1 cytochrome *b5* -like domain demonstrated that the PGRMC1-bound heme iron is five-coordinated, leaving one open face, and that PGRMC1 Y113 serves as the axial iron-coordinating residue (Kabe et al., 2016). To test whether axial iron coordination is required for PGRMC1’s ability to bind and stabilize cytochromes P450, we mutated PGRMC1 tyrosine 113 to phenylalanine (Y113F). PGRMC1 Y113F still bound heme and functioned like wild-type PGRMC1 (McGuire et al., 2021). A second mutant PGRMC1 (Y113F, K163A, Y164F) that does not bind heme also bound and stabilized a cytochrome P450. Thus, PGRMC1 binding to heme is not required for its observed role in supporting cytochromes P450 stability and activity (McGuire et al., 2021). Interestingly, the PGRMC1 Y113F mutant was not fully functional. PGRMC1 binds to mitochondrial ferrochelatase, the final enzyme in heme synthesis (Piel et al., 2016). Mutation of Y113F completely disrupted binding of PGRMC1 to ferrochelatase in mouse liver. Together, these results indicated that PGRMC1 Y113 axial coordination of the heme iron is required for ferrochelatase binding, but not binding and stabilization of cytochromes P450 (McGuire et al., 2021).

Treatment of fungal cells with cobalt chloride inhibits ergosterol synthesis, and cells with defects in ergosterol synthesis fail to grow on cobalt chloride containing medium (Lee et al., 2007). Fission yeast Dap1 binds both Erg5 and Erg11 and is required for their function in ergosterol synthesis (Hughes et al., 2007). Consequently, *dap1Δ* cells fail to grow on cobalt chloride containing medium (Hughes et al., 2007). Previously, we demonstrated that Dap1 heme binding is required for cytochromes P450 function in ergosterol synthesis because disruption of heme binding to Dap1 phenocopied a *dap1Δ* mutant. Here, we tested whether axial coordination of heme iron is required for Dap1 function and whether Dap1 stabilizes cytochromes P450 like mammalian PGRMC1.

Protein sequence alignments revealed strong sequence homology in the cytochrome b5-like domains of PGRMC1 and Dap1. PGRMC1 Y113 corresponds to Dap1 Y83 (Figure Panel A). To test whether axial coordination of the heme iron is required for Dap1-dependent cytochromes P450 function, we assayed the ability of different *dap1* mutants to rescue growth of *dap1Δ* cells on medium containing cobalt chloride. Wild-type cells, but not *dap1Δ* cells, grew on cobalt chloride medium (Figure Panel B, columns 1 and 2). Plasmid-based expression of wild-type *dap1 ^+^* rescued growth of *dap1Δ* cells. Mutation of Dap1 Y83 to either histidine or phenylalanine is predicted to alter iron coordination. Neither *dap1 Y83H* nor *dap1 Y83F* rescued growth on cobalt chloride (Figure Panel B, columns 4 and 5), indicating that proper Dap1 axial coordination of heme iron is required for cytochromes P450 function in fission yeast.

PGRMC1 functions to stabilize cytochromes P450 in mouse liver and cultured cells (McGuire et al., 2021). To test if this is also true in fission yeast, we assayed the expression of the two cytochrome P450 enzymes, Erg5 and Erg11, in the presence and absence of Dap1. Expression of Dap1, Erg5, and Erg11 was detected using different epitope tags that were encoded at the 3’ end of the endogenous coding sequence. Each epitope tagged protein was functional since cells expressing *dap1-13xMyc erg5-3xHA erg11-GFP* grew normally on cobalt chloride containing medium. Expression of each protein was detectable by western blotting (Figure Panel C, lane 2). Expression of Erg5 and Erg11 was not altered in *dap1Δ* cells compared to wild-type cells (Figure Panel C, compared lanes 2 and 3). Additional studies indicated that Erg11 is a stable protein with a half-life longer than a cell division cycle (>2 hours), and that deletion of *dap1 ^+^* had no effect on Erg11 half-life. These experiments indicate that Dap1 does not function to stabilize cytochromes P450 in fission yeast.

To summarize, PGRMC1 is required for cytochromes P450 stability and activity in mouse liver, but this does not require PGRMC1 Y113 axial coordination of heme iron or heme binding (McGuire et al., 2021). Conversely, mutation of the axial ligand in Dap1 Y83 phenocopies deletion of *dap1 ^+^* , and Dap1 is not required for cytochromes P450 stability. How can we reconcile these differences between mammalian PGRMC1 and fission yeast Dap1? One possibility is that the two proteins do not share a conserved function. The fission yeast cytochrome P450 proteins Erg5 and Erg11 may be stable and not regulated at the level of protein turnover. In addition, while PGRMC1 robustly binds ferrochelatase (McGuire et al., 2021; Piel et al., 2016), we did not detect binding of Dap1 to ferrochelatase in affinity purification mass spectrometry experiments (Hughes et al., 2007). If we assume that there is a conserved function, we speculate that both PGRMC1 and Dap1 may bind to ferrochelatase and function to deliver heme to cytochromes P450. In yeast, ferrochelatase binding (undetected to date) and heme transport may require axial heme iron coordination. In mouse liver, PGRMC2 may compensate for the loss of PGRMC1 and supply heme to cytochromes P450. PGRMC1 may additionally function to stabilize cytochrome P450 enzymes. Detailed mechanistic studies in both systems are required to resolve these questions.

Given the brevity of this report, it is important to note limitations to the study and our data interpretations. In making our conclusions, we assumed that Dap1 Y83F still binds to heme and that only axial iron coordination is disrupted as is the case for PGRMC1 Y113F. We have not assessed iron coordination or heme binding *in vitro* for Dap1 Y83H or Dap1 Y83F. Further, we have not directly assayed Erg5 and Erg11 activity, but rather we used sensitivity to growth on medium containing cobalt chloride as an indirect assay for ergosterol synthesis.

## Methods

Yeast were grown to log phase at 30°C in rich YES medium (0.5% [w/v] yeast extract plus 3% [w/v] glucose and supplements, 225 μg/ml each of uracil, adenine, leucine, histidine, and lysine). *S. pombe* strains were derived from the wild-type haploid KGY425 using homologous recombination and standard genetic techniques (Bahler et al., 1998; Burke and Gould, 1994; Moreno et al., 1991). Plasmids were generated by site-directed mutagenesis, sequence verified, and transformed into yeast (Alfa et al., 1993). Yeast transformants were screened for expression of 3xHA-Dap1 by western blotting as previously described (Hughes et al., 2005; Shao et al., 2020). Clones with equal expression were selected for phenotypic analysis.

## Reagents

**Table:** 

**STRAIN**	**GENOTYPE**	**SOURCE/REFERENCE**
KGY425	*S. pombe* , *h−, his3-D1, leu1-32, ura4-D18, ade6-M210*	ATCC
PEY901	*S. pombe* , *h−, dap1-D1::kanMX6, his3-D1, leu1-32, ura4-D18, ade6-M210*	Espenshade Lab, Hughes et al. 2007
PEY1070	*S. pombe* , *h* + *, dap1-13xMyc::kanMX6, erg5-3xHA::kanMX6, erg11-GFP::kanMX6, his3-D1, leu1-32, ura4-D18, ade6-M210*	Espenshade Lab, this study
PEY1072	*S. pombe* , *h−, dap1-D1::kanMX6, his3-D1, leu1-32, ura4-D18, ade6-M210*	Espenshade Lab, this study
		
**PLASMID**	**DESCRIPTION**	**SOURCE/REFERENCE**
pSLF101	CaMV promoter expression vector carrying *S. cerevisiae LEU2*	Addgene, Forsburg 1993
pPJE602	* 3xHA-dap1 ^+^* in pSLF101	Espenshade Lab, Hughes et al. 2007
pAH57	*3xHA-dap1 Y83H* in pSLF101, nucleotides 247-249 (TAT -> CAT)	Espenshade Lab, this study
pSZ1	*3xHA-dap1 Y83F* in pSLF101, nucleotides 247-249 (TAT -> TTT)	Espenshade Lab, this study
		
**ANTIBODY**	**ANIMAL AND CLONALITY**	**SOURCE; WORKING CONCENTRATION**
Anti-Myc	Mouse monoclonal 9E10	Santa Cruz, sc-40; 1:1000
Anti-HA	Rabbit polyclonal HA.11	Covance, Catalog # PRB-101P; 1:1000
Anti-GFP	Mouse monoclonal (clones 7.1 and 13.1)	Roche Catalog # 1814460; 0.4 mg/ml

Standard chemicals were obtained from Sigma or Thermo Fisher Scientific. We obtained yeast extract from BD Biosciences; amino acids from Q-Biogene; oligonucleotides from Integrated DNA Technologies; and IRDye 800CW or IRDye 680RD-conjugated goat anti-mouse or anti-rabbit secondary IgG from LI-COR. Plasmid pSLF101 was obtained from Addgene (Forsburg, 1993). Sources for primary antibodies are listed in the table.
